# Correlates and impact of DSM-5 binge eating disorder, bulimia nervosa and recurrent binge eating: a representative population survey in a middle-income country

**DOI:** 10.1007/s00127-022-02223-z

**Published:** 2022-01-19

**Authors:** Jose C. Appolinario, Rosely Sichieri, Claudia S. Lopes, Carlos E. Moraes, Gloria V. da Veiga, Silvia Freitas, Maria A. A. Nunes, Yuan-Pang Wang, Phillipa Hay

**Affiliations:** 1 Obesity and Eating Disorders Group, Institute of Psychiatry, Federal University of Rio de Janeiro, Av. Professor Gastão Bahiana, 496 ap1809, Rio de Janeiro, 22071-030 Brazil; 2 Social Medicine Institute, State University of Rio de Janeiro, Rio de Janeiro, Brazil; 3 Nutrition Institute, Federal University of Rio de Janeiro, Rio de Janeiro, Brazil; 4 State Institute of Diabetes and Endocrinology, Rio de Janeiro, Brazil; 5 Federal University of Rio Grande do Sul, Porto Alegre, Brazil; 6 Instituto de Psiquiatria (LIM-23), Universidade de São Paulo, São Paulo, Brazil; 7 Translational Health Research Institute, Western Sydney University, Penrith, Australia; 8 Camden and Campbelltown Hospitals, SWSLHD, Campbelltown, Australia

**Keywords:** Binge eating disorder, Bulimia nervosa, Recurrent binge eating, Prevalence, Middle-income country

## Abstract

**Purpose:**

Binge eating disorder (BED), bulimia nervosa (BN) and recurrent binge eating (RBE) are binge eating spectrum conditions causing a significant impact in individual’s health and functioning. Information regarding those conditions came mostly from high-income countries. The objective of this study was to assess the prevalence of DSM-5 BED, BN and RBE and correlates in a representative sample from a metropolitan area of a middle-income country.

**Methods:**

The data were obtained from a cross-sectional population-based household survey in two stages in Rio de Janeiro, Brazil. Noninstitutionalized residents aged 18–60 years were assessed by lay interviewers using the Questionnaire of Eating and Weight Patterns-5 (QEWP-5). Positive cases and a paired sample screen-negative cases were reassessed by phone with the Eating Disorders Section of SCID-I-P (adapted for DSM-5). The data were collected from September 2019 to February 2020.

**Results:**

Overall, 2297 individuals were interviewed. Prevalence of BED was 1.4%, BN 0.7%, RBE 6.2%. Psychiatric comorbidities, such as depression, anxiety and ADHD were significantly more prevalent in people with BED, BN and RBE than in people without these eating problems. Several medical conditions, when controlling for body mass index, were significantly more prevalent in people with BED, BN and RBE. People with BED and BN had marked impairments in work/school, social and family life, reduced mental and physical HRQoL and under half had sought treatment.

**Conclusion:**

As in high income countries, in Rio de Janeiro, Brazil, BED, BN and RBE are prevalent conditions and are associated with elevated BMI, functional impairment, psychiatric and medical comorbidity and poorer HRQoL.

## Introduction

Binge eating is characterized by the consumption of large amounts of food in a short period of time accompanied by feelings of loss of control over eating [1]. Binge eating is a core diagnostic component of eating disorders (ED), such as binge eating disorder (BED) and bulimia nervosa (BN) in DSM-5 [2] and ICD-11 [3]. BED is characterized by recurrent and distressing episodes of binge eating not followed by compensatory behaviors. It is considered the most prevalent ED in the general population with an estimated point prevalence ranging from 0.6 to 3.6% [4] and is increasing [5]. It commonly presents in individuals with a high body mass index (BMI; kg/m^2^), but it is not limited to them [6]. BN is also defined by the same recurrent episodes of binge eating seen in BED, but these are followed by inappropriate compensatory behaviors to prevent weight gain. It is also characterized by an overvaluation of weight and/or shape. BN point prevalence in the community is 0.6% [7, 8]. BED and BN are severe mental disorders frequently associated with psychological and nonpsychological problems, impairment in functionality and health related quality of life (HRQoL).

The concept of binge eating spectrum conditions is increasingly employed in the literature and usually encompasses BED, BN and partial syndromes, such as recurrent binge eating (RBE) [9, 10]. Although there is no final consensus with respect of a unique definition of RBE, it would be conceptualized as episodes of binge eating occurring at-least in a weekly basis, but not fulfilling criteria for a full ED diagnosis. Recently, several studies suggested that people with RBE are prevalent in the community [11–13]. In addition, RBE has been associated with psychological distress [14], metabolic disorders, such as overweight, obesity and metabolic syndrome [15], poorer HRQoL and functional disability [16]. It is also considered an escalating health problem. Mitchison et al. [12] found a sixfold increase in the general population prevalence of RBE from 1998 (2.7%) to 2015 (13.0%) in Australia. Thus, it would be appropriate to also assess the impact of RBE along with the formal ED diagnosis when studying the impact of binge eating spectrum conditions in the population.

Despite the public health impact of those conditions, a review on ED epidemiology [17] concluded that the vast majority of population-based data of BED, BN and RBE come from high-income countries and there is a paucity of information regarding other parts of the world. Furthermore, it was thought that BED prevalence in low- and middle-income Latin America countries could be higher than that observed in Western regions [4]. As BED occurs more frequently in individuals with elevated BMI, this could be a concomitant phenomenon of the high rates of overweight and obesity observed in countries like Brazil [18] and potentially associated with some socioeconomic inequalities [19].

There have been a series of preliminary studies assessing the broad concept of RBE in the Brazilian population. Freitas et al. [20] carried out a study in a population sample in Rio de Janeiro, where they evaluated 1295 women aged 35 years or more. They found a 11.5% prevalence of RBE (twice a week) in the previous 6 months. De França et al. [21] using a time frame of 3 months found a prevalence of recurrent binge eating (twice a week) of 2.7%. Obesity, fair/poor self-rated health status and body dissatisfaction were strongly associated with binge eating status. The largest study that investigated BED and BN diagnosis in a representative general population was the WHO World Mental Health Survey which included a Brazilian sample from São Paulo—the São Paulo Mega City Health Survey [22]. Using the Composite International Diagnostic Interview (CIDI), trained lay interviewers assessed 2942 subjects. The authors found, in the metropolitan area of São Paulo, a life-time prevalence of 4.7 and 2.0% and a 12-month estimate of 1.8% and 0.9% for DSM-IV BED and BN, respectively.

Furthermore, there is little known about the physical and mental health burden and health service use of people with binge eating spectrum conditions in Brazil. Udo and Grilo [23] have reported high rates of medical comorbidity (particularly cardiovascular and metabolic disease) for people with BED but not BN in a large representative US sample. The authors commented it also important to investigate these associations controlling for BMI. Also, important to investigate is the health service use of people with these disorders as research has found a severe treatment gap [24] which may be greater in lower income regions of the world.

The aim of this two-phase, household survey was to assess the point prevalence of DSM-5 BED, BN and RBE in a representative sample of noninstitutionalized residents of the city of Rio de Janeiro, Brazil. We also aimed to investigate other associated features, such as psychiatric and clinical comorbidity, functional impairment, work productivity, HRQoL and treatment status in a major metropolitan city of Brazil.

## Methods

### Design and sample

Binge eating in Rio was designed as a survey of the general population of the city of Rio de Janeiro. Rio de Janeiro, or Rio, is the capital of the State of Rio de Janeiro and the second-largest city of Brazil with an estimated population for 2021 of 6,775,561 (https://www.ibge.gov.br/) [25]. Rio is also the 6th largest city in the Americas. As other big cities in middle-income countries, Rio has several inequalities, such as income distribution, housing, health, transportation and violence. Over one third of Rio’s population (2.5 million) live in favelas [26]. Rio’s inhabitants represent a microcosm of Brazil’s ethnic diversity and include people of European, African and mixed ancestry.

The study was an in-person household survey with two phases. Adults aged 18–60 were included. Women who were pregnant and breast feeding were excluded. A sample size was required for estimation of at least 13% prevalence of BED, BN and RBE [12, 27, 28]. The survey was based on a stratified and clustered probability sample selected in three stages: census enumeration areas (CEAs), households and eligible adults (18–60 years old). The primary sample units (153 CEAs) were stratified according to the 31 administrative regions of the city and the mean household income of CEAs and were selected with probability proportional of its number of households in the previous 2010 Population Census. In the second stage, 20 households were selected in each CEA using an inverse sample procedure [29, 30]. In the third stage, an eligible adult was selected with equal probability among the household eligible residents. The sample size to estimate a prevalence of at least 2%, with relative error of 45%, confidence level of 95% and a design effect of 2.4, reach 2232 adults in the sample. No estimate of nonresponse was used, since the inverse sample in each CEA finishes when the fixed number of households interviewed is reached.

The participants were selected at random from each of the households where there was more than one individual in the required age range. The data from the Brazilian Demographic Census conducted by IBGE [25] served as the basis for the sample design. The study was approved by the research ethics committee of the Institute of Psychiatry, Federal University of Rio de Janeiro. All study participants provided a written informed consent.

## Procedures

After sample selection, each selected household were contacted by the interviewers and invited to participate in a survey about their eating habits and mental health status. The potential participants also received a letter, signed by the Principal Investigator presenting the study objectives and introducing the work team. At the first visit, and after verifying availability, household was confirmed, and the interviewer scheduled the interview. If availability was not confirmed either new visits were conducted or a refusal was registered. Respondents were not paid for their participation in the survey.

The data were collected in two phases from September 1, 2019 until February 21, 2020. In phase 1 from the 2985 eligible households 688 refused to participate and the final participation rate was 77%. A total of 2297 individuals were enrolled. In this phase, participants were invited by trained lay interviewers to complete a research questionnaire adapted to a computer-assisted device (tablet). They also measured weight and height. In phase 2, participants who screened positive and a sub-sample of screen-negative cases in QEWP-5 were invited to answer a telephone interview for diagnosis confirmation.

## Measures

### Binge eating spectrum conditions

#### Phase 1

BED, BN and RBE were assessed by the *Questionnaire on Eating and Weight Patterns*-5. QEWP-5 is a self-report instrument developed for the screening of BED and BN according to DSM-5 criteria [31]. For the purposes of this study RBE meet the description of a binge according to DSM 5 as well as the frequency criteria for BED—binge eating at least once a week for 3 months. Conversely, RBE differs from DSM-5 BED because it does not include the binge eating associated features and the criterion of marked distress regarding the episode.

The validity of the Brazilian Portuguese version of QEWP-5 used in this study for the assessment of BED, BN and RBE was evaluated in comparison with the Structured Clinical Interview for DSM-IV (SCID-P) (adapted for the DSM-5) in a subsample of study participants [32]. For the general screening of ED (BED, BN and their subthreshold forms), QEWP-5 showed a sensitivity of 0.75, a specificity of 0.70, a positive predictive value of 0.67 and a negative predictive value of 0.77. The final questions of the survey asked the age at onset (year) and treatment information.

#### Phase 2

A research assistant selected all screen-positive cases of BED, BN and their subthreshold forms. For each case, the assistant randomly selected paired screen-negative participants. A total of 305 (13.2%) participants from phase 1 were selected to phase 2. Those participants were contacted 1–2 weeks after the field interview and were interviewed by two doctoral students, trained in the application of SCID-I-P and with large experience with EDs (CEM, CM) using the EDs Section of the SCID-P [33] (adapted for the DSM-5). The SCID-P is a clinical interview and it was considered our gold standard method for diagnosis. The application of SCID-P by telephone has been widely used in ED [34, 35] area and has been validated in comparison to face-to-face interviews. All interviews were reviewed and discussed with a senior expert investigator (JCA) for case confirmation.

#### Socio-demographics

The following socio-demographic characteristics of participants were collected: gender, age, self-defined race/ethnicity, marital status, schooling, employment status, income.

#### Treatment

Treatment was provided by the Brazilian health system known as SUS (Sistema Unico de Saude). Patients do not pay for their medical appointments or hospital visits. Approximately 80% of the population relies on SUS for healthcare and approximately 25% of people hold private policies [36]. To assess treatment search we asked the following questions’: “Did you seek treatment for these episodes of overeating you described?” If the answer was “yes”, we asked in the sequence “What kind of professional did you search? with the following alternatives: “(a) general practitioner, (b) psychiatrist, (c) psychologist, a (d) dietitian, (e) others.

#### Psychiatric comorbidity

A validated Brazilian version of the Patient Health Questionnaire (PHQ-9) [37] was used as a diagnostic instrument for self-reported depressive symptoms. It is a multiple-choice instrument consisting of nine items based on the diagnostic criteria for major depression of the DSM-IV. In the present study, depression was defined by a PHQ-9 score of 10 or higher, which is considered as the presence of clinically relevant depressive symptoms [38]. For anxiety screening, the GAD-7 instrument validated in Brazil was used [38]. The GAD-7 is a 7-item questionnaire designed to identify probable cases of anxiety and measure the severity of symptoms. Generalized anxiety symptoms was defined by a cutoff of 8 or more [39, 40]. For the screening of symptoms of Attention-Deficit Hyperactivity Disorder (ADHD), we used the World Health Organization (WHO) adult attention-deficit/hyperactivity disorder self-report screening scale for DSM-5 (ASRS-Screening Scale) [41]. Symptoms of ADHD was defined with a ASRS with cut-off point ≥ 14. Alcohol-related problems was assessed using the AUDIT (Alcohol Use Disorders Identification Test) an instrument developed by the World Health Organization (WHO) AUDIT was used to screen for alcohol-related problems [42]. This study employed the Brazilian version of this questionnaire in 2005 [43]. Its use has proved important for the adequate screening and diagnosis of alcohol-related problems in the general population. Alcohol use was defined by an AUDIT cutoff point > 8.

#### Medical comorbidity

The questions about medical comorbidities (chronic diseases and injuries related to ED) were adapted from the National Health Survey [44]. We collected information related to hypertension, diabetes mellitus, heart disease, cerebrovascular accident (stroke)/stroke, asthma, arthritis and work-related musculoskeletal diseases (WRMSDS). In addition, the data on other clinical conditions associated with ED [23], such as pain syndromes (e.g., headache, cervical and lumbar pain, arthritis, myalgias and arthralgias and fibromyalgia) and gastrointestinal syndromes (gastroesophageal reflux disease and irritable colon syndrome) were collected with the use of specific questions. For the evaluation of medical comorbidity, the following question was asked, based on the self-report of medical diagnosis: (a) “Has any doctor ever given you a diagnosis of (disease X)?”.

### Functional impairment, work productivity

BED, BN and RBE functional impairment was measured with the Sheehan Disability Scale (SDS) [45]. SDS is a composite of three self-rated items of work, social and family life impairment. The SDS also measures the number of workdays lost and the number of underproductive workdays. A study of the psychometric properties of the SDS have demonstrated the validity, reliability and sensitivity to change in individuals with eating disorders [46] and other psychiatric conditions [47]. We used a Brazilian version of the instrument currently in use in Brazil [48].

#### HRQoL

The SF-12 [49], a short version of the Medical Outcomes Survey (MOS) SF-36 was used to assess HRQoL. Silveira et al. [50] studied the psychometric properties of the SF-12 in the Brazilian population. The Physical (PCS) and Mental Component Scores (MCS) presented averages respectively, equal to 49.6 (SD = 9.0) and 51.9 (SD = 8.6). Furthermore, Cronbach's alpha coefficient (*α* = 0.836) presented a high degree of reliability.

#### Anthropometry

BMI (kg/m2) was calculated based on the current weight and height measured by trained interviewers. Subjects' weights were measured using a digital scale with a maximum capacity of 150 kg and a precision of 100 g (Plenna^®^, São Paulo, Brazil). They were weighed at home while barefoot, wearing light clothing and standing at the center of the scale, with arms hanging alongside the body. Height was measured using a portable stadiometer with a maximum range of 200 cm and a precision of 0·1 cm (model 206; Seca^®^, Hamburg, Germany).

### Statistical analysis

Weighted prevalence and respective 95% confidence intervals of BED, BN and RBE were estimated. Weights adjust for differences in the probability of selection and for nonresponse, considering the probability of selection in each of the sampling stages, including within-household selection. For all analysis, the effect of the complex design of the survey was accounted for using “Proc Survey” procedures with Taylor series variance estimation in the Statistical Analysis System (SAS) (release 9.5) [51].

Cross-tabulations of BED, BN and RBE were computed for prevalence of comorbid psychiatric, clinical conditions and quality of life. Demographics and metabolic characteristics according to ED status were tested by Wald chi-square test based on the difference between observed and expected weighted frequencies. Bivariate and multiple logistic regression models were used to calculate the prevalence ratios with Poison distribution, allowing estimation of relative risk (RR)s. Confounding variables BMI, gender and race/skin color were included in the models based on the literature and testing of the association in the sample for psychiatric and clinical comorbidity. Scores of functionality and quality of life were tested by t tests.

All statistical analysis including 95%CI were estimated considering weights and the complex design of the survey. Models 2 were adjusted for BMI, gender and race.

## Results

### Prevalence and socio-demographics

We found a point prevalence of 1.4% for BED, 0.7% for BN, and 6.2% for RBE. Participants with BED, BN and RBE had a mean age of 40.3 (SE = 3.3), 31.9 (SE = 3.7) and 34.7(SE = 1.4) years. Compared to the general population, BED, BN and RBE were significantly more prevalent in females 83.2%, 90.1% and 76.5% and in individuals with black ethnicity for BN 49.0% (Table 1).

**Table 1:** Prevalence of eating disorder status, according to demographics and metabolic characteristics of the population in Binge Eating in Rio Survey (*n* = 2297), 2020

Variables	BED			BN			RBE		
	*n*	%	95% CI	*n*	%	95% CI	*n*	%	95% CI
Total	29	1.4	0.81–2.43	17	0.7	0.34–1.55	90	6.2	3.10–5.27
Gender									
Male	**5**	**0.5**	**0.18–1.34**	**2**	**0.1**	**0.03–0.62**	**19**	**2.4**	**1.40–3.99**
Female	**24**	**2.3**	**1.21–4.19**	**15**	**1.3**	**0.56–2.86**	**71**	**5.6**	**4.12–7.58**
Race/skin color									
White	6	0.8	2.91–2.12	6	0.5	0.18–1.20	34	3.6	2.35–5.41
Black	7	2.4	0.91–6.31	**6**	**1.8**	**0.56**–**5.90**	18	4.2	2.15–7.90
Mixed^a^	16	1.5	0.79–2.79	5	0.5	0.17–1.22	38	4.4	3.06–6.30
Age									
18–30 years	6	1.1	0.44–2.86	5	1.0	0.25–3.91	32	5.3	3.45–8.19
31–45 years	12	1.3	0.67–2.41	10	1.0	0.41–2.44	33	3.8	2.50–5.66
46–60 years	11	1.8	0.75–4.44	2	0.1	0.03–0.66	25	3.1	1.76–5.14
Marital status									
Single	10	1.5	0.54–4.18	4	0.4	0.12–1.15	40	4.6	3.14–6.80
Married	16	1.3	0.67–2.60	9	1.0	0.37–2.48	42	4.0	2.70–6.02
Widow/divorced	3	1.5	0.34–6.11	4	0.7	0.25–2.23	8	1.9	0.89–4.04
Schooling^a^									
0–10 years	10	1.4	0.54–3.60	7	1.0	0.31–3.34	37	4.4	2.70–7.09
11–14 years	15	1.6	0.76–3.54	9	0.7	0.28–1.96	42	4.1	2.75–5.99
> 15 years	4	0.7	0.24–2.56	1	0.1	0.01–0.74	11	3.2	1.4–7.1
Income^c^									
Up to R$1.000.00		1.4	0.58–3.56	5	1.6	0.41–6.27	21	3.9	2.23–6.87
R$1.001.00–3.000.00	15	1.8	0.77–4.37	9	0.8	0.29–2.12	36	4.0	2.64–6.06
> R$3.000.00	5	0.7	0.25–1.71	–	–	–	14	3.4	1.75–6.45
BMI^d^									
Underweight	0	–		0	–	–	3	7.0	01.67–24.86
Normal weight	**1**	**8.1**	**1.08–41.64**	**2**	**7.7**	**1.60–30.06**	16	18.2	**10.56**–**29.51**
Overweight	**6**	**27.1**	**9.13–57.88**	**4**	**18.2**	**4.04–53.97**	24	21.8	**13.50**–**33.15**
Obese	**21**	**64.8**	**36.12–85.68**	**11**	**74.1**	**40.54–92.32**	46	53.0	**40.21**–**65.51**

Values in bold indicate statistically significant at a* p*-value < 0.05

*BED* binge eating disorder (DSM-5), *BN* bulimia nervosa (DSM-5), *RBE* recurrent binge eating (≥ 1 binge eating episode/week in the last 3 months), *BMI* body mass index

^a^Mixed: Brown, Yellow and Indigenous

^b^Income = R$1000.00 is approximately one minimum wage in Brazil

^c^Schooling = 0–10 years (equivalent to elementary), 11–14 years (equivalent to high school), > 15 years of study (college or above)

^d^BMI = weight (KG)/height (m2)

### Clinical characteristics and treatment search

Based on the DSM-5 severity specifiers, 56.1% (SE = 10.5) of BED cases were considered mild, 22.0% (SE = 3.0) were rated moderate and 21.9% (SE = 10.1) were severe or extreme. BED participants had a late mean Age of Onset (AOO) of 33.1 years (SE = 3.7) with 10.5 years (SE = 2.5) since the onset of their condition. Only 42.4% (SE = 13.5) individuals with BED had sought treatment. From those who sought professional help 35.3% (SE = 17.8) contacted a dietitian, 16.3% (SE = 11.6) a general practitioner and, 25.9% (SE = 16.0) a mental health professional (psychologist or a psychiatrist). For BN, 51.3% (SE = 16.5) of participants were classified as mild and 48.7% (SE = 16.5) as moderate in terms of DSM-5 severity specifiers. Subjects with BN had a mean AOO of 22.9 (SE = 3.0) years and had 9.06 (SE = 3.9) years since the disease onset. For BN also, only 44.7% (SE = 17.4) of subjects sought a treatment for their condition, 29.2% (SE = 19.9) sought a dietitian or a GP and 21.6% (SE = 10.8) a mental health professional. In the case of RBE subjects, the mean AOO was 26.24 (SE = 3.0) years with a mean of 8.2 (SE = 1.2) years since the onset. Only 19.5% (SE = 5.7) of the subjects with RBE sought treatment, 72.2% (SE = 29.1) contacted a dietitian or a GP and only 16% (SE = 10.2) a mental health professional.

### Psychiatric comorbidity

High proportions of individuals with BED, BN and RBE screened positive for depression (PHQ-9), anxiety (GAD-7), ADHD (ASRS-6) and alcohol use disorder (AUDIT) (Table 2). The relative risk (RR) for depression, anxiety and ADHD symptoms was higher in participants with BED and BN than in those without an ED. Although with lower figures, subjects with RBE also had significant RRs of depression, anxiety and ADHD. In addition, only BN was associated with higher risk for alcohol use. Further multivariate regression adjusting for sex, race and BMI status did not change the results.

**Table 2:** Number of cases (*n*), prevalence (%), relative risk (RR) and 95% CI (confidence interval) of psychiatric comorbidity according to eating disorder status in the Binge Eating in Rio survey (*n* = 2297), 2020

Psychiatric comorbidity	BED *n* = 29					BN *n* = 17					RBE *n* = 90				
	Model 1			Model 2		Model 1			Model 2		Model 1			Model 2	
	*n* (%)	RR	95% CI	RR	95% CI	*n* (%)	RR	95% CI	RR	95% CI	*n* (%)	RR	95% CI	RR	95% CI
Depression (PHQ-9)ª	21 (81.7)	**8.1**	**6.4–10.3**	**7.1**	**5.3–9.6**	13 (82.9)	**7.9**	**6.1–10.1**	**6.9**	**4.9–9.9**	35 (29.7)	**2.9**	**1.9–4.3**	**2.8**	**1.9–4.3**
Anxiety (GAD-7)^b^	22 (76.9)	**7.1**	**5.5–9.2**	**5.8**	**4.3–7.7**	10 (63.1)	**5.5**	**3.4–9.1**	**4.5**	**2.8–7.4**	39 (30.3)	**2.8**	**1.9–4.0**	**2.6**	**1.7–3.9**
ADHD (ASRS-6)^c^	12 (35.8)	**8.8**	**4.1–18.7**	**10.3**	**4.3–24.7**	8 (54.3)	**13.1**	**6.1–28.1**	**13.8**	**6.8–28.0**	17 (19.0)	**4.9**	**2.8–8.6**	**4.9**	**2.7–9.0**
Alcohol use (AUDIT)^d^	5 (15.9)	2.0	0.6–6.0	0.9	0.3–2.8	4 (23.5)	**5.3**	**2.2–12.8**	**6.3**	**2.6–15.1**	12 (12.2)	1.5	0.8–3.0	1.2	0.6–2.4

Model 1 Univariate Poisson regression; Model 2 Poisson regression adjusted by BMI, gender, race/skin color

Calculations of Relative Risk and 95% confidence intervals were adjusted for survey weights

Values in bold indicate statistically significant at a* p*-value < 0.05

*BED* binge eating disorder (DSM-5), *BN* bulimia nervosa (DSM-5), *RBE* recurrent binge eating (≥ 1 binge eating episode/week in the last 3 months). *PHQ-9* Patient Health Questionnaire-9, *GAD-7* 7-item Generalized Anxiety Disorder Scale, *ADHD* attention-deficit/hyperactivity disorder, *ASRS* Adult ADHD Self-Report Scale, *AUDIT* Alcohol Use Disorder Identification Test

^a^Depression: Assessed using a PHQ-9 with cutoff point ≥ 10

^b^Anxiety: Assessed using a GAD-7 with cutoff point ≥ 10

^c^ADHD: Assessed using a ASRS with cutoff point ≥ 14

^d^Alcohol use: assessed by AUDIT cutoff scores = a score of > 8

### Medical comorbidity

BED was associated with a greater number of self-reported medical conditions (Table 3). Individuals with BED had a significantly likelihood of having diabetes, asthma, arthritis/rheumatism, spine problems, chronic headache, chronic muscle pain and gastroesophageal reflux compared to those without an ED condition. These results remained significant after controlling for gender, race and BMI in a multivariate regression analysis. BN was associated with a higher risk for hypertension, chronic headache and chronic muscle pain, and RBE with spine problems and chronic muscle pain. However, only hypertension in BN remained significant after controlling for confounders using multivariate analysis.

**Table 3:** Number of cases (*n*), prevalence (%), relative risk (RR) and 95% CI of self-reported medical conditions, according to eating disorder status in Binge Eating in Rio Survey (*n* = 2297), 2020

Clinical comorbidity^a^	BED *n* = 29					BN *n* = 17					RBE *n* = 90				
	Model 1			Model 2		Model 1			Model 2		Model 1			Model 2	
	*N* (%)	RR	95% CI	RR	95% CI	*N* (%)	RR	95% CI	RR	95% CI	*N* (%)	RR	95% CI	RR	95% CI
Arterial hypertension	9 (38.0)	2.5	0.8–7.6	1.7	0.6–5.4	4 (7.1)	0.31	0.82–1.16	**0.2**	**0.04–0.61**	24 (19.8)	1.0	0.56–1.78	0.7	0.4–1.3
Diabetes	10 (38.4)	**9.3**	**3.6–24.1**	**7.6**	**3.0–19.4**	0 (–)	–	–	-	-	4 (3.0)	0.46	0.15–1.43	0.4	0.1–1.2
Heart disease^c^	1 (3.0)	0.6	0.1–4.7	0.5	0.1–4.1	0 (–)	–	–	-	-	7 (4.2)	0.84	0.32–2.18	0.7	0.3–1.7
Stroke	1 (2.1)	1.5	0.2–11.0	1.0	0.1–7.3	0 (–)	–	–	-	-	2 (1.5)	1.0	0.23–4.32	0.8	0.2–3.3
Asthma	5 (29.2)	**5.5**	**1.69–17.5**	**5.4**	**1.9–15.9**	2 (30.6)	5.8	0.97–35.15	4.8	0.9–26.3	7 (6.9)	0.98	0.36–2.61	0.5	0.2–1.1
Arthritis/rheumatism	6 (12.2)	**3.1**	**1.1–8.9**	**2.6**	**1.0–6.6**	0 (–)	–	–	–	–	2 (1.3)	0.29	0.07–1.21	0.3	0.1–1.1
Spine problems^c^	17 (56.2)	**6.3**	**2.0–19.7**	**4.2**	**1.6–11.3**	6 (22.1)	1.4	0.38–5.08	1.1	0.3–3.9	11 (27.3)	**1.85**	**1.1–3.2**	1.7	1.0–3.0
WMSDs^d^	3 (6.0)	2.1	0.5–8.9	1.9	0.5–7.9	1 (3.3)	1.1	0.13–8.98	1.0	0.1–8.5	3 2.0	0.64	0.18–2.29	0.6	0.2–2.2
Chronic headache	11 (41.0)	**5.0**	**2.3–11.3**	**4.3**	**1.5–12.4**	8 (34.3)	**3.8**	**1.01–14.2**	2.4	0.6–9. 2	21 (17.7)	1.56	0.91–2.68	1.2	0.7–2.1
Chronic muscle pain	17 (53.0)	**6.2**	**2.1–18.4**	**3.5**	**1.4–8.4**	8 (43.6)	**4.2**	**1.15–15.7**	2.6	0.6–11.2	26 (26.1)	**1.94**	**1.14–3.3**	1.7	1.0–2.8
Fibromyalgia	2 (4.5)	4.9	0.8–28.8	3.0	0.6–15.6	1 (2.7)	2.9	0.38–22.7	1.5	0.2–14.2	0 (–)	–	–	–	–
Gastroesophageal reflux	7 (16.9)	**5.0**	**1.6–15.8**	**4.7**	**1.6–13.7**	3 (6.6)	1.75	0.46–6.7	1.4	0.4–5.2	8 (8.3)	2.3	0.89–5.7	2.1	0.9–5.1
Irritable bowel syndrome	0 (–)	–	–	–	–	1 (3.3)	3.2	0.42–24.7	2.0	0.2–17.3	2 (2.5)	2.5	0.6–10.4	1.9	0.5–8.3

Model 1—Univariate Poisson regression; Model 2—Poisson regression adjusted by BMI, gender, race/skin color. Calculations of Relative Risk and 95% confidence intervals were adjusted for survey weights

Values in bold indicate statistically significant at a* p*-value < 0.05

*BED* Binge Eating Disorder (DSM-5), *BN* Bulimia Nervosa, *RBE* Recurrent Binge Eating—(≥ 1 binge eating episode/week in the last 3 months)

^a^This variable was assessed with the question: "Has any doctor ever given you a diagnosis of (disease X)?"

^b^Heart disease (myocardial infarction, angina, heart failure or others)

^c^Spine problems such chronic back or neck pain, lumbago, sciatic pain, vertebra or disc trouble

^d^Work-related musculoskeletal disorders (WMSDs)

### Functionality, work productivity

Overall, BED and BN participants showed moderate/marked impact in functionality in all domains of SDS (Table 4). Functional impairment in work/school area were significantly greater for BED (mean 6.2) and BN (mean 4.6) than for RBE (mean 0.6). BED and BN individuals displayed a significant impairment in social life, (mean 7.1) and (mean 6.9), respectively; compared to RBE (mean 2.3). Family life of BED (mean 6.7) and BN (7.0) individuals was also significantly impacted compared to those with RBE (mean 2.4).

**Table 4:** Means and standard error (SE) of functionality, work productivity and quality of life according to eating disorder diagnosis in binge eating in Rio survey (*n* = 2297), 2020

	BED *n* = 29			BN *n* = 17			RBE *n* = 90			*p* value^1^ BED vs noED BN vs noED RBE vs noED	*p* value BED vs BN BED vs RBE RBE vs BN
	Mean	SE	95% CI	Mean	SE	95% CI	Mean	SE	95% CI		
Work/school	6.2	1.2	3.85–8.57	4.6	0.9	2.90–6.36	2.5	0.6	1.27–3.66	**–**	0.26
											**0.0045**
											**0.04**
Social life	7.1	1.4	4.32–9.94	6.9	1.4	4.08–9.68	2.3	0.7	0.96–3.73	**–**	0.86
											**0.002**
											**0.008**
Family life	6.7	1.2	4.23–9.28	7.0	1.4	4.22–9.69	2.4	0.6	1.18–3.68	**–**	0.88
											**0.002**
											**0.005**
Days lost	3.3	0.8	1.68–4.95	4.1	1.1	1.92–6.25	0.8	0.3	0.20–1.35	**–**	0.51
											**0.005**
											**0.004**
Days impaired	4.2	1.2	1.83–6.63	4.4	1.0	2.36–6.53	0.9	0.3	0.39–1.493	**–**	0.83
											**0.009**
											**0.002**
Quality of Life (SF-12)											
Physical health	**44.4**	**1.9**	**40.45–48.29**	**46.6**	**2.9**	**40.77–52.46**	**51.2**	**1.0**	**49.11–53.23**	**< 0.0001**	0.51
										**0.02**	**0.0 03**
										**0.01**	0.15
Mental health	**29.9**	**2.1**	**25.70–34.16**	**29.0**	**1.7**	**25.45–32.48**	**40.5**	**1.8**	**37.03–44.09**	**< 0.0001**	0.73
										**< 0.0001**	**0.0002**
										**0.001**	**< 0.0001**

Values in bold indicate statistically significant at a* p*-value < 0.05

*BED* Binge Eating Disorder (DSM-5), *BN* Bulimia Nervosa, *RBE* Recurrent Binge Eating—(≥ 1 binge eating episode/week in the last 3 months)

^1^*t* test

Participants with BED and BN incurred significantly more workdays lost (mean 3.3 and 4.1, respectively) from their normal daily responsibilities and more underproductive days at school or work (mean 4.2 and 4.4 days, respectively), compared with those with RBE (mean 0.8 days lost, 0.9 days impaired) (Table 4).

### HRQoL

Mental and physical HRQoL based on the SF-12 mental and physical component scores were significantly lower for participants with BED, BN and RBE than those without an ED (Table 4). Comparing the different ED rubrics in a post hoc analysis, BED and BN showed a significantly greater impact in physical component of HRQoL as compared to RBE. This impact in the Mental Health component of SF-12 was even greater for BED and BN in comparison with BED.

## Discussion

The present study based on a representative sample of a major city in Brazil provides unique data on BED, BN and RBE. In the best of our knowledge, it is the first major epidemiological survey to report the prevalence of binge eating spectrum conditions based on DSM-5 criteria in a middle-income country. In general, our results confirm that BED, BN, RBE in Brazil are prevalent conditions, highly associated with psychiatric and medical comorbidity. Furthermore, as observed in high-income countries BED and BN had a significant impact in the individual daily life (functional impairment and HRQoL) [52, 53].

The DSM-5 prevalence of 1.4% for BED and 0.7% for BN observed in this study are in line with the figures reported in other community-based surveys [54, 55]. However, two other population-based studies using the same two-stage methodology found a lower 12-month prevalence of DSM-5 BED and BN of 0.44 and 0.14 in US [56] and a 3.6 and 0.7 in UK [57], respectively. Several, differences in study design and methods (e.g. use of instruments that employed a ‘skip-out’ function and did not assess all symptoms) [56] could explain these discrepancies [17]. In addition, the current study was conducted in a highly urbanized area with high levels of socio-economic disadvantage that may increase ED risk [58]. Importantly, our findings are also consistent with the overall prevalence for BED and BN reported in the WHO World Mental Health Survey [22]. Although this study was based on DSM-IV criteria, the authors reported a 12-month prevalence of 1.8 for BED and 0.9 for BN in the city of São Paulo. Considering the clinical relevance of RBE as a potential precursor of a full-fledged ED we found that 6.2% of the population in Rio de Janeiro displayed this behavior in accordance with the numbers reported by other authors [12, 21]. BED, BN and RBE were significantly more frequent in females and BN more frequent in black participants.

It is noteworthy that, according to the DSM-5 severity criteria, 43.4% of the subjects with BED in our sample were classified as moderate to extreme cases with a late mean AOO (33.1 years). Furthermore, 42% of BED subjects sought a treatment for their condition and only 25% of them have searched a mental health professional. As expected, participants with BN had an earlier mean AOO of 22.9 years and 48.7% of the cases were considered of moderate degree of severity. In the case of BN, from the 44.7% of subjects who sought treatment, less than 10% contacted a mental health professional. Of note, Kessler et al. [22] reported quite similar AOO for BN (20.6 years), but a lower AOO for BED (23.3 years). Although there are few population-based data on EDs DSM-5 severity levels, the proportion of moderate to extreme cases observed in our study was higher for BED and BN than observed in a community sample in US [59, 60]. As observed in other countries [22, 24], less than a half of individuals with BED and BN seek some form of treatment, but the most striking here is the low proportion of those contacting a mental health professional, consistent with the findings of Hay et al. [61].

Like other epidemiological surveys [22, 23], a high number of subjects with binge eating spectrum conditions displayed significant symptoms of depression, anxiety, ADHD (and alcohol use in the case of BN) compared with the general population. Interestingly and less explored yet, we found also similar patterns of ADHD comorbidity in BED and BN. Our results also demonstrate the high degree of association of BED with several medical conditions, such as diabetes, asthma, arthritis/rheumatism, spine problems, chronic headache, chronic muscle pain and gastroesophageal reflux independent of the weight status.

A high rate of functional impairment has been reported in BED and BN [62, 63]. We found also an overall and quite similar levels of functional impairment in subjects with BED and BN in our study. In addition, BED and BN, but not RBE, were associated with a marked impairment in the three domains of SDS (work/school, social and family life) with similar figures found in other severe mental disorders [64]. Work productivity was also impacted in BN and BED individuals as reported by Pawaskar et al. [63] in a population-based survey in US.

A systematic review summarizing the results of 42 studies investigating the quality of life in EDs concluded that patient’s HRQoL was significantly worse in BN and BED when compared with healthy populations [65]. Subjects with BN, BED and RBE showed significantly more impairment than controls in both physical and mental health domains of SF-12. However, the magnitude of this impact in the mental health domain was higher and quite similar for BED and BN when compared to RBE.

The major strengths of this study are: (1) the use of a large epidemiological dataset with a representative sample of a major city in a middle-income country, (2) the use of a two-stage epidemiological design, (3) a comprehensive assessment of binge eating spectrum correlates, such as psychiatric/medical comorbid conditions, functionality, work productivity and quality of life and (4) the use of regression models to analyze the associations of binge eating spectrum and psychiatric/clinical comorbidity and quality of life controlling for the effect of confounders, such as higher BMI, sex and race.

The main limitation of the study was the low specificity for detecting the diagnostic categories of BED and BN and moderate specificity for detecting the whole spectrum of binge eating conditions of the QEWP-5 [32] which increases the likelihood of false positives. Another point is that the population of this study was not representative of the less urbanized Brazilian population. For example, Souza et al.[66] investigated the effect of living in rural and urban areas on the prevalence of depression in a representative sample of the Brazilian population. They found that the prevalence of depression was lower in rural areas, when compared with urban areas. Last, the psychiatric comorbidity was assessed only with screening instruments and not using diagnostic interviews, again which may have led to false positives.

Our findings have several clinical and public health implications. The confirmation that BED and BN are prevalent, underdiagnosed and undertreated conditions in the country can help health care providers/legislators in planning future strategies and policies to improve the awareness and management of those disorders. Another important point is that about half of subjects with BED and BN in our sample was actively seeking some health advice and, of those, the great majority contacted a dietitian or a GP. As addressed by Monteleone et al. [67] this calls the attention for the need of a multiprofessional strategies in the recognition and management of these individuals. Our data also suggested that RBE, is a prevalent phenomenon and, albeit to a lesser degree than BED or BN, there were associations with psychiatric comorbidity and HRQoL impairments. Thus, as demonstrated by other authors [12, 21], RBE could serve as a marker of binge eating spectrum disorder and be included in the screening in the usual clinical practice.

Furthermore, the information provided by this survey provides, to our knowledge, the first general population information regarding the burden of BED and BN in the developing world. A recent publication [68] found the consideration of BED in the 2019 iteration of the Global Burden of Diseases study more accurately informs policy makers worldwide about the high burden experienced by people living with these disorders and the need to address this burden.

Future research should delineate the syndrome of RBE further (e.g. with consideration to specifiers of functionality and/or psychological distress) and investigate aspects related to the course of binge eating spectrum conditions longitudinally in low- and middle-income countries. In conclusion, like in high-income countries, BED, BN and RBE are prevalent conditions in the city of Rio de Janeiro, Brazil. These eating disturbances are significantly associated with elevated BMI, psychiatric and medical comorbidity, poor HRQoL and functional impairment in the case of BED and BN.

## Availability of data

The data that support the findings of this study are available from the corresponding author (JCA) upon reasonable request.
